# Do trialists endorse clinical trial registration? Survey of a Pubmed sample

**DOI:** 10.1186/1745-6215-8-30

**Published:** 2007-10-23

**Authors:** Ludovic Reveiz, Karmela Krleža-Jerić, An-Wen Chan, Sylvia De Aguiar

**Affiliations:** 1 Sanitas Research Institute, Fundación Universitaria Sánitas, Bogotá, Colombia; 2 Canadian Institutes of Health Research (CIHR), Ottawa, Ontario, Canada; 3 Department of Medicine, University of Toronto, Toronto, Ontario, Canada; 4 Latin American Ongoing Clinical Trial Register (LATINREC), Bogotá, Colombia

## Abstract

**Introduction:**

Despite intense interest in trial registration, there is a wide gap between theoretical postulates on trial registration and its implementation worldwide.

**Objective:**

We aimed to evaluate trialists views about current international guidelines on trial registration, including the World Health Organization's (WHO) International Clinical Trials Registry Platform (ICTRP) policies and the Ottawa Statement, as well as their intention to register any future clinical trials they conduct.

**Methods:**

We identified all 40,158 PUBMED-indexed clinical trials published from May 2005 to May 2006 using an advanced search strategy. From a random sample of 500 confirmed clinical trials, corresponding authors with e-mail contact addresses were surveyed.

**Results:**

A total of 275 (60%) questionnaires from 45 countries were completed. 31% of the respondents had received only nonindustry funding during the past ten years, while 5% and 61% had received only industry or mixed funding respectively. Approximately two third of participants supported registration of all 20 WHO Data Set items, and endorsed the Ottawa Statement part 1 and part 2. Delayed public disclosure of some essential data in instances where they may be considered sensitive for competitive commercial reasons was supported by 30% of the participants, whereas immediate disclosure was supported by 53%. Only 21% of participants had registered all of their ongoing trials since 2005, while 47% stated that they would provide the 20 WHO Data Set items to a publicly accessible register for all their future clinical trials; a significantly higher proportion of participants who received only nonindustry funding (62%) was found among those who would always provide the 20 WHO items for future trials, compared to 42% of participants who received mixed or only industry funding. Among those who were undecided about endorsing registration. One third of participants expressed a lack of sufficient knowledge as the primary reason.

**Conclusion:**

Although disagreement was apparent on certain issues, our findings illustrate that trial registration is gradually becoming part of the current research paradigm internationally. Our results also suggest that researchers require more knowledge to inform their decision to comply with the International standards at this early stage of voluntary trial registration.

## Background

Over the past few years, several key initiatives have enabled substantial progress in the public registration of clinical trials worldwide. Major registers such as ClinicalTrials.gov and Controlled Clinical trials-ISRCTN have been recording trial protocol information since 2000, and the number of registered trials increased dramatically in 2005 after the requirement for registration was introduced by several medical journals, led by the International Committee of Medical Journal Editors (ICMJE) [1,2]. The World Health Organization's International Clinical Trials Registry Platform (WHO ICTRP) is currently leading international efforts to implement global standards for trial registration and results reporting [2-4]. Based on both ethical and scientific reasons for trial registration, WHO is urging investigators, research institutions and companies to register all trials that prospectively test the effects of healthcare interventions on humans.

Furthermore, the Ottawa Group, an international group of individuals and organizations from the international medical research community, developed two consensus documents – the Ottawa Statement Part 1 on the principles of trial registration, and the Ottawa Statement Part 2 on the principles of its implementation The Ottawa group supported the WHO initiative as a good initial step, but called for more details of trial protocols to be prospectively registered [5-7]. The Institute of Medicine report endorses the ICJME requirement to prospectively register clinical trials as a condition for publication, as well as the pharmaceutical industry's commitment to register and post clinical trials results [8].

Nevertheless, despite intense interest in this topic in recent years, there is a wide gap between theoretical postulates on trial registration and its implementation worldwide. This gap may be due to differing interests of the various stakeholders involved, including researchers, patients, pharmaceutical industry, funders, governments, registries, bio-medical journal editors, and the public [9-11]. One of the biggest barriers to comprehensive trial registration is the lack of awareness by researchers about the importance of the problem [9]. According to the Ottawa Statement part 1, "the principal investigator has a responsibility to ensure that the sponsor(s) obtains a Unique Identification number and registers his or her contact information, the protocol information and the trial results" [5].

Although there is general agreement about the type of protocol information that should be registered for a trial, as defined by the 20-item WHO Registration Data Set (Table 1) [11,1], controversy surrounded issues related to registration of key versus all secondary outcomes, as well as immediate versus delayed disclosure of 5 protocol items that were felt to be commercially sensitive by industry for some trials (study interventions, scientific title, sample size, primary and secondary outcomes) [10]. Industry has offered, instead, to consign these five hidden items to a locked electronic depository that is publicly inaccessible until the information is no longer deemed commercially sensitive [10]. Regarding results reporting on trial registration, the principles expressed in the Ottawa Statement 1 read "At a minimum, results for outcomes and analyses specified in the protocol (as approved by the IRB/IEC), as well as data on harms, should be registered regardless of whether or not they are published. If a trial is terminated prematurely, any available results should be registered along with the reason for termination." [5,6]. However, the Ottawa Group stated that researchers should have sufficient time to publish their findings before the registered results are released for public [5].

**Table 1:** Minimum data set that should be recorded for clinical trial registration, according to the International Standards launched by the World Health Organization, in 2006 (items felt to be commercially sensitive are highlighted)

**Number**	**Item**	Abbreviated Definition/Explanation*
1.	Primary register trial number	Name of Primary Register, and the unique ID number assigned by the Primary Register to this trial.
2.	Trial registration date	Date when trial was officially registered in the Primary Register.
3.	Secondary IDs	Other identifying numbers and issuing authorities besides the Primary Register, if any
4.	Source(s) of monetary or material support	Major source(s) of monetary or material support for the trial
5.	Primary sponsor	The individual, organization, group or other legal entity which takes responsibility for initiating, managing and/or financing a study.
6.	Secondary sponsor(s)	Additional individuals, organizations or other legal persons, if any, that have agreed with the primary sponsor to take on responsibilities of sponsorship.
7.	Contact for public queries	Email address, telephone number, or postal address of the contact who will respond to general queries, including information about current recruitment status
8.	Contact for scientific queries	Email address, telephone number, or postal address, and affiliation of the person to contact for scientific queries about the trial
9.	Public title (of the study)	Title intended for the lay public in easily understood language.
**10.**	**Scientific title**	**Scientific title of the study as it appears in the protocol submitted for funding and ethical review**
11.	Countries of recruitment	The countries from which participants will be, are intended to be, or have been recruited.
12.	Health condition or problems studied	Primary health condition(s) or problem(s) studied
**13.**	**Intervention(s)**	**Specific name of the intervention(s) and the comparator/control(s) being studied**
14.	Key inclusion and exclusion criteria	Inclusion and exclusion criteria for participant selection, including age and sex.
15.	Study type	A single arm study is one in which all participants are given the same intervention. Trials in which participants are assigned to receive one of two or more interventions are NOT single arm studies. Crossover trials are NOT single arm studies.
		A trial is "randomized" if participants are assigned to intervention groups using a method based on chance (e.g., random number table, random computer-generated sequence, minimization, adaptive randomization).
16.	Date of the first enrollment (anticipated or actual date of the enrollment of the first study participant)	If the trial is being registered after recruitment of the first participant record actual date of Anticipated date of enrollment of the first participant.
**17.**	**Target sample size**	**Number of participants that this trial plans to enroll.**
18.	Recruitment status	Recruitment status of the trial.
**19.**	**Primary outcome(s)**	**Outcomes are events, variables, or experiences that are measured because it is believed that they may be influenced by the intervention. The Primary Outcome should be the outcome used in sample size calculations, or the main outcome(s) used to determine the effects of the intervention(s).**
**20.**	**Key secondary outcomes**	**Secondary outcomes are events, variables, or experiences that are of secondary interest or that are measured at timepoints of secondary interest.**

* Full items definition and/or explanation can be viewed in the International Clinical Trials Registry Platform (ICTRP) website (25).

The aim of our study is to evaluate trialists' views about the global standards proposed by WHO and the Ottawa Statements, as well as their intention to register any future clinical trials they conduct.

## Materials and methods

We used PUBMED to identify a sample of investigators authoring clinical trial reports published from May 2005 to May 2006. The structured search filter included the following terms: "Randomized controlled trial" [PT] OR "controlled clinical trial" [PT] OR "randomized controlled trials" [MeSH] OR "random allocation" [MeSH] OR "double-blind method" [MeSH] OR "single-blind method" [MeSH] OR "clinical trial" [PT] OR "clinical trials" [MeSH] OR "clinical trial" [TW] OR "randomized trial" [TW] OR "randomised trial" [TW]. The strategy focused on humans studies and was not limited by language or type of intervention.

For this exploratory survey, we identified 40,158 references, from which a random sample of 500 clinical trials with e-mail contact addresses was selected. Titles and abstracts identified from the sample were screened by one reviewer (LR). A previous study showed that approximately 25% of articles found by the search are clinical trials and cite the e-mail of one author [12]. The sample size was calculated assuming that 5% of emails would not reach their intended recipient. In addition, Web-based surveys have variable response rates (9 to 96%), depending on the topic, methods and participants [13,14]. The *a priori *response rate was estimated to be 75%. In the absence of previous data, we estimated the proportion of trialists who would endorse registration to be 50%, yielding a sample size of 500 with a 95% confidence interval of -5% to 5%. The primary outcomes of our study were the proportion of trialists endorsing the following principles: trial registration overall, registration of all secondary outcomes, immediate public disclosure of registered protocol items, and public disclosure of trial results.

The survey was piloted on 50 participants. For the full sample, each participant was sent a survey by email containing links to the WHO ICTRP and the Ottawa Group websites [see Additional file 1]. Up to four reminders were sent. We collected the following information: country of origin, gender, age, type of funding for clinical trials over the past 10 years, main research institution, number of ongoing trials, proportion of ongoing trials registered in public databases, knowledge and support for the WHO Registration Data Set, as well as attitudes towards the Ottawa Statements (part 1 and part 2), registration of key versus all secondary outcomes, timing of public disclosure of protocol items, and disclosure of trial results.

Data were analyzed using SPSS 12.0. Chi-square tests were used to determine associations between categorical variables.

## Results

### Demographic data

Out of 500 e-mail questionnaires, forty-two (8%) email addresses were invalid (bounced back or out-of-office autoreply). We received 275/458 (60%) replies from 45 countries. The median age of participants was 45 years (10th-90th percentiles: 33 – 57) and 70% were men. The largest number of respondents was from the United States (15%), Italy (9%), United Kingdom (6%), France (5%) and Germany (5%). However 20% of participants did not include their country of origin in the survey, which made the evaluation of response rate by country difficult.

Of the 275 (60%) survey respondents, 31% received only non-industry funding over the past ten years (Table 2). Type of funding reported by participants was significantly different between women and men; 44% of women received non industry funding compared to 27% of men while 50% and 68% received mixed funding and 6% and 5% received only industry funding respectively (X^2 ^= 7.82; DF 2, p = 0.02). Most respondents were based at an academic or hospital institution (Table 2).

**Table 2:** Characteristics of respondents N = 275

		Total	Women n = 82 30%	Men n = 193 70%
**Source of trial funding received over the past ten years**	Only nonindustry	85 (31%)	44%	27%
	Only industry	14 (5%)	6%	5%
	Mixed	168 (61%)	50%	68%
	No response	8 (3%)		
**Main institution of respondents**	Academic	154 (56%)		
	Hospital	90 (33%)		
	Governmental	6 (2%)		
	Industry (eg. pharmaceutical, device, biologics, etc)	3 (1%)		
	Other type	14 (5%)		
	No response	8 (3%)		

### Trial registration issues

One third of trialists stated that they were experts or very knowledgeable about issues related to trial registration, while 56% reported having mild to moderate knowledge; 12% reported having no relevant knowledge.

64% of investigators supported registration of all 20 items of the WHO Data Set, while 6% did not support any. 65% and 63% already had or planned to endorse the Ottawa Statements (part 1 and part 2) respectively; 3% and 3% did not support the statements, while 31% and 34% respectively were undecided. The attitudes of respondents were similar regarding the Ottawa Statements and the WHO standards.

**Figure 1:**
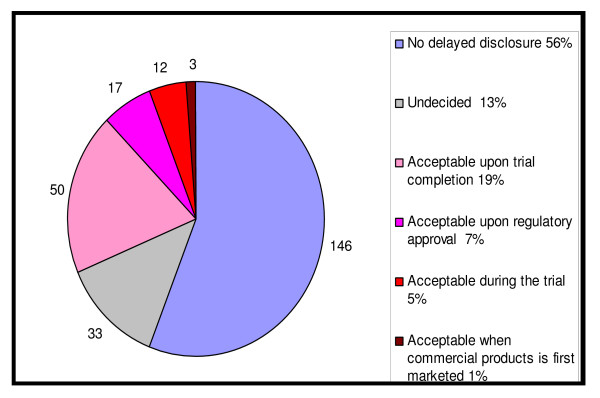
Position of respondents regarding a delayed disclosure of five essential protocol items n = 261.

Ninety-five trialists (35%) believed that all secondary trial outcomes should be registered and disclosed, whereas 139 (51%) stated that only key secondary outcomes should be registered and disclosed. With regards to timing of disclosure for five essential data items (Scientific title, Intervention(s), Target sample size, Primary outcome(s), and Key secondary outcomes) in instances where they may be considered sensitive for competitive reasons, no delayed disclosure was supported by 53% of trialists while 31% supported delayed disclosure Among those who agreed with delayed disclosure in some cases, most considered that undisclosed items should be made publicly available upon trial completion or upon regulatory approval (Figure 1). The Ottawa Group position with regards to registration of trial results was completely or mostly supported by 61% of participants, while 10% were not supportive at all.

The median number of ongoing clinical trials per respondent was 3 (10th-90th percentile range: 0 – 7). Only 21% of participants had registered all of their ongoing trials. In addition only 47% of respondents stated that they would provide all 20 items of the WHO Data Set to a publicly accessible register for future clinical trials.

Between respondents receiving only non-industry funding compared to those receiving only industry or mixed funding, there were no significant differences for the proportion endorsing trial registration overall, registration of secondary outcomes, delayed disclosure of protocol items, or results registration. A significantly higher proportion of participants who received only nonindustry funding (62%) was found among those who would always provide the 20 WHO items for future trials, compared to 42% of participants who received mixed or only industry funding (X^2 ^= 8.59; df 1, p = 0.0034).

57% of participants did not provide their names; no significant difference regarding registration of future trials was found among anonymous respondents compared to those who included their names (X^2 ^= 12.4; df 7, p = 0.087). However more women (70%) were anonymous compared to men (47%) (X^2 ^= 6.03; df 1, p = 0.014).

### Summary of trialists' concerns regarding trial registration

We received a wide range of open comments. Common concerns were expressed related to (a) the lack of time to complete "bureaucratic" tasks (4%), (b) the possibility that registration of early (phase I) trials could deter research as their results may be misinterpreted (2%), and (c) that the results of poorly-designed trials might be confusing (1%). Three trialists expressed concern that the Ottawa Statement or the WHO initiative offers no protection from other competing investigators who may copy the trial, recruit faster and publish sooner. In addition, they mentioned the administrative burden of handling additional public queries as well as contractual obligations to sponsors would be difficult to handle. Other participants stated that some countries have limited internet access, and that registration should be a tool for protecting the patient's rights rather than yet another means of over-burdening researchers.

## Discussion

### Main findings

Our survey provides useful information about trialists' attitudes towards clinical trial registration. Although more than 60% of respondents support both the WHO 20-item Data Set and the Ottawa Statements (parts 1 and 2), only 47% declared that they would always provide the WHO Data Set to a publicly accessible register for future clinical trials. In addition a third of participants were undecided regarding trial registration while a small minority rejected it outright.

The proposal by WHO and the Ottawa Group to submit protocol information at trial inception as well as results to a freely accessible public register is a crucial step towards promoting research transparency. However, countries have different legal requirements and most do not require public registration of all clinical trials. They also have diverse levels of public disclosure of information. For example all entities conducting clinical trials of experimental treatments for serious or life-threatening conditions or diseases in the United States (US) are required to submit certain information to the ClinicalTrials.gov register, which is a publicly accessible database. However the US legislation does not include other type of diseases, and does not require the registration of all 20 items suggested by WHO. Furthermore, information on Phase I trials of drugs and devices is not publicly available unless they have been approved by the US Food and Drug Administration [15]. It is evident that there is a need for comprehensive legislation on trial registration. Otherwise, when ongoing or completed trials remain hidden, researchers may be unknowingly and inappropriately duplicating trials on similar interventions that had already been shown in previous trials to have serious adverse events or no benefit.

Our survey demonstrated greater support for immediate rather than delayed disclosure of information submitted to trial registers (53% vs. 30%), Figure 1. However, some researchers argue that confidentiality issues and contractual obligations with sponsors would be difficult to handle if they decided to register all clinical trials in a publicly accessible register. Some stakeholders also claim that disclosure of all 20 WHO data set items may sometimes endanger proprietary rights. However others argue that delayed disclosure would facilitate registration of incomplete information for a given trial, and hidden trial information would not meet ethical and scientific standards [4,16-18]. In addition, trial participants are already informed about a given study as part of the informed consent process, and intelligence companies provide detailed data about pipeline drugs at a cost; thus this information is not secret [19,20]. Furthermore, informed choice about which particular trial to join requires that information about all ongoing trials be available to each potential trial participant.

A universal requirement for clinical trial registration as a condition of ethics approval would level the playing field and address concerns over competitive disadvantage [21]. In the interim, voluntary registration of clinical trial information remains an important first step, and dissemination strategies to inform researchers about trial registration are thus needed, particularly in developing countries.

More participants supported registration and disclosure of only key rather than all secondary outcomes. Use of the adjective "key" introduces subjectivity in its definition, as outcomes may be considered as non-key by some researchers but essential by others.

## Limitations

Some concerns have been raised regarding Web based surveys including coverage bias or bias due to sampled individuals not having or choosing not to access the Internet [22-24]. There was selection bias due to less than half of the authors having e-mail addresses listed in the publication. For example there were 8 publications from Russia but no electronic addresses were available. Furthermore a substantial percentage of those with valid email addresses did not respond. There are also limitations related to PubMed. Although the MEDLINE database indexes more than 3500 biomedical journals, it excludes many existing trials and authors worldwide. In addition PUBMED may be biased toward English-language journals and has poorer coverage of European journals than compared to other databases. Considering that 74% of respondents were from non-English speaking countries, there was unlikely to be language bias from the survey being in English.

It should also be noted that there were few participants from commercial or government institutions.

At the same time, participants who had some knowledge about trial registration may have been more able to respond. It is highly probable that a number of respondents may have needed more knowledge about trial registration to answer specific questions, although we provided links to relevant information on the Internet.

It is disappointing that although 64% of participants endorsed trial registration, only 47% stated that they would always provide at least all 20 WHO Data Set items for future trials. This finding may reflect their concerns over academic or commercial interests.

## Conclusion

Selective reporting of information about ongoing and completed trials is harmful for society as it violates ethical and scientific responsibilities, and distorts the body of evidence available for clinical decision-making. Voluntary registration of clinical trial information is an important and complex initial step that has the support of almost half of trialists. Dissemination strategies to inform researchers about the process and benefits of trial registration are needed to improve compliance.

## Competing interests

Ludovic Reveiz is currently the Director of the Latin American Clinical Trial Register (Latinrec), which is part of a non profit Organization that aim to freely register clinical trials in Latin America. Karmela Krleza-Jeric is leading the Ottawa group and is a member of the Scientific Advisory Board (SAG) of the WHO International Clinical Trials Registry Platform (ICTRP). An-Wen Chan is a member of the Ottawa Group editorial and was formerly a scientist with the ICTRP, WHO.

## Authors' contributions

All the authors conceived the study and participated in the design. AWC, KK and LR contributed to the questionnaire design. SA administrated electronic questionnaires and entered the data. LR analyzed the data. All authors contributed to drafting the manuscript and read and approved the final manuscript.

## Supplementary Material

Additional file 1 Survey. Survey provided to respondents. Click here for file
