# Supplementary material for: Do trialists endorse clinical trial registration? Survey of a Pubmed sample
Source: Trials. 2007 Oct 23;8:30. doi: 10.1186/1745-6215-8-30 (PMC2147029; doi:10.1186/1745-6215-8-30)
Supplement: Additional file 1 — Survey. Survey provided to respondents. [file 1745-6215-8-30-S1.doc]

**Dear Dr,**

 We are writing to invite your contributions to a survey assessing trialists’ views of the [**International Clinical Trials Registry Platform**](http://www.who.int/ictrp/en/) **(ICTRP) of the World Health Organization (WHO)** **and of the Ottawa Statement (** [**http://ottawagroup.ohri.ca**](http://ottawagroup.ohri.ca/)**) .** We chose your name from a random sample taken from Medline; your contact details were obtained from this source too.

(**Go to survey:** [**http://cico.com.co/clinicaltrial_02_2006/**](http://cico.com.co/clinicaltrial_02_2006/))

       The primary objectives of the (**ICTRP**) are "to ensure that all clinical trials are registered and thus publicly declared and identifiable, so as to ensure that for all trials, a minimum set of results will be reported and made publicly available". By registering an on-going trial, trialists are encouraged to provide general information and particular details about the methods of the study protocol. This will address a long awaited development; to systematically register clinical trials at their inception in a publicly and universal accessible register.

 The Ottawa Group is an independent grass-roots   organization of interested stakeholders that has been conducting a worldwide dialogue on trial registration. It developed the Ottawa Statement Part 1 on  the  principles of trial registration that has been published in BMJ, posted on the Ottawa Group website, and  endorsed by about 150  individuals and groups. The Ottawa Statement part 2 on the principles of implementation of trial registration is currently open on [http://ottawagroup.ohri.ca](http://ottawagroup.ohri.ca/) for comments and endorsements.

        We shall be grateful if you would contribute to our short electronic survey (will take you about **5-8 minutes** to complete) and share your opinions on the principles and implementation of trial registration. We are providing you with links to additional sources of information for your perusal.

 Your answers will remain confidential and will be used for the statistical analysis but specifics will not be reported as to single out any participant.

**Go to survey:** [**http://cico.com.co/clinicaltrial_02_2006/**](http://cico.com.co/clinicaltrial_02_2006/)

| **Survey on Trial Registration Issues** |
| --- |

Top of Form

| 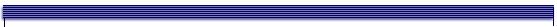 | |
| --- | --- |
| 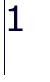 | Gender  Female     Male |
| 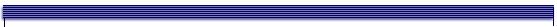 | |
| 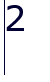 | Age  years |
| 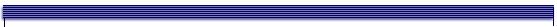 | |
| 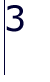 | Main Research Institution   |  | Academic | | --- | --- | |  | Industry (eg. pharmaceutical, device, biologics, etc) | |  | Hospital | |  |  | |  | Government | |  | Other | |
| 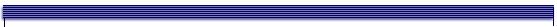 | |
| 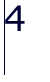 | Trial registration refers to the recording of trial information on a public database so that its existence is known.  How knowledgeable do you feel about issues related to trial registration?  a- no knowledge  b- little knowledge  c- average knowledge  d- Very knowledgeable  e- expert |
|  |  |
| 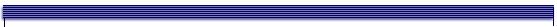 | |
| 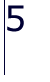 | The [**Ottawa Statement**](http://ottawagroup.ohri.ca/statement.html) (Part 1) recommends prospective (upfront) registration of all trials, public disclosure of standard key trial details, registration of any subsequent amendments, as well as registration and public disclosure of all results.   Would you consider endorsing the Ottawa Statement part 1 (OS 1)?   |  | Yes, already did | | --- | --- | |  | Yes, will do at http://ottawagroup.ohri.ca/join.html | |  | Undecided | |  | No | |
| 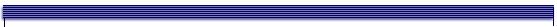 | |
| 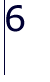 | If undecided or no, please rate your level of disagreement with the principles described in the Ottawa Statement part 1:  a- No disagreement with the principles  b- Disagree with a few principles  c- Disagree with some principles  d- Disagree with most principles  f- Disagree with all principles  **Please list your main reasons for disagreement:**   |  |  | | --- | --- | |  |  | |
| 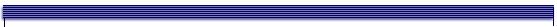 | |
| 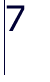 | Principles of implementation of trial registration are proposed in a draft of the Ottawa Statement (Part 2). These include the assignment of a globally unique identification number, definition of trial data items for registration, and criteria for acceptable registers and a search portal.  **Would you consider endorsing the Ottawa Statement (Part 2)?**   |  | Yes, already did | | --- | --- | |  | Yes, will do at http://ottawagroup.ohri.ca/join.html | |  | Undecided | |  | No | | **If undecided or no, please rate your level of disagreement with the principles described in the Ottawa Statement (Part 2):**  a- No disagreement with the principles  b- Disagree with a few principles  c- Disagree with some principles  d- Disagree with most principles  f- Disagree with all principles | | |  | | |  |  | |  |  | |  |  |   **Please list your main reasons for disagreement** |
| 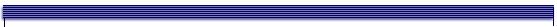 | |
| 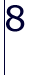 | What is your level of knowledge about the [**International Clinical Trials Registry Platform of the World Health Organization**](http://www.who.int/ictrp/en/) (WHO)?  a- Expert knowledge  b- Very knowledgeable  c- Moderately knowledgeable  d- Mildly knowledgeable  e- I have never heard of the WHO Registry Platform |
| 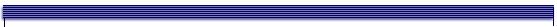 | |
| 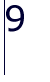 | According to the WHO Registry Platform, the following minimum Data Set should be recorded in a publicly accessible register prior to participant recruitment:   - Primary register trial number - Trial registration date - Secondary IDs - Source(s) of monetary or material support - Primary sponsor - Secondary sponsor(s) - Contact for public Queries - Contact for Scientific Queries - Public title (of the study) - Scientific title - Countries of Recruitment - Health Condition or Problems Studied - Intervention(s) - Key inclusion and exclusion criteria - Study type - Date of the first enrolment (Anticipated or actual date of the enrolment of the first study participant) - Target sample size - Recruitment status - Primary outcome(s) - Key secondary outcomes   **Do you support registration of the above 20 items (WHO Data Set)?**   | a- I do not support registering any of the 20 items | | --- | | b- I support registering **a few** of the 20 items | | c- I support registering **some** of the 20 items | | d- I support registering **most** of the 20 items | | e- I support registering **all** of the 20 items | | f- Undecided |   **If you are not supportive of registering all 20 items, please explain**:  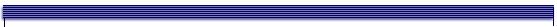  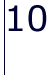  The WHO Registry platform proposes to register only KEY Secondary Outcomes.  Some people feel that outcomes which are worth studying are also worth registering, ie all outcomes specified in the protocol should be registered and disclosed.  Others believe that only “key” outcomes should be registered and disclosed.  **Which statement best reflects your view on this issue?**   1. All secondary outcomes should be registered and disclosed 2. Only key secondary outcomes should be registered and disclosed 3. Undecided 4. Other: ____________________________   Please provide three most important reasons for you choice: |
| 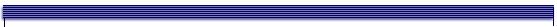 | |
| 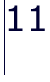 | After registering trial information, some stakeholders have asked for delayed public disclosure of some or all of the following five data items in instances where they may be considered sensitive for competitive commercial reasons:  • Scientific title  • Intervention(s)  • Target sample size  • Primary outcome(s)  • Key secondary outcomes  The Ottawa group disagrees with delayed public disclosure of any of the 20 items because it feels that it would compromise research transparency.  **What is your view with regards to the timing of public disclosure of the above five items after registration?**  1- The 5 items should be publicly disclosed immediately upon registration for all trials.  2- Delayed disclosure of some or all the 5 items should be allowed in some cases.    3- I am undecided on this issue    4- Other:________________________________  **If you agree with delayed disclosure in some cases, when should the items be made publicly available?**   During the trial   Upon trial completion   Upon regulatory approval   When the intervention is first marketed   Other: _______________ |
|  | |
| 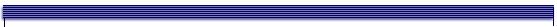 | |
| 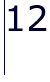 | The Ottawa Group also recommends that TRIAL RESULTS should be registered and publicly disclosed once the analyses are completed and verified.  However investigators should have sufficient time to publish their findings in a peer-reviewed electronic or print forum before the registered results are released for public, free-of-charge access.   Do you support the Ottawa Group position with regards to trial results registration?   |  | Not supportive at all | | | --- | --- | --- | |  | A little supportive | | |  | Somewhat supportive | | |  | Mostly supportive | | |  | Completely supportive | | |  | Undecided |  | |
| 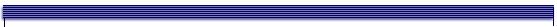 | |
| 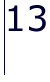 | Please indicate the number of ongoing clinical trials (still recruiting patients) in which you are currently involved: |
| 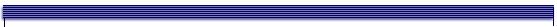 | |
| 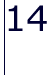 | **If you are currently involved in ongoing clinical trials:**  How many of your ongoing trials are already registered in a publicly accessible register?   |  | All | | --- | --- | |  | Most | |  | Some | |  | Few | |  | None | |  | Unsure | |  | I am not involved in any ongoing trials | |
| 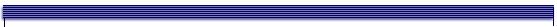 | |
| 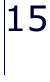 | For future clinical trials in which you are an investigator, will you provide at least the 20 items (WHO Data Set) to a publicly accessible register?   |  | Never | | --- | --- | |  | Rarely | |  | Sometimes | |  | Most of the time | |  | Always | |  | Undecided |   **If not always, please explain:** |
| 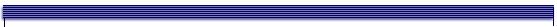 | |
| 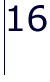 | Please provide any anonymous comments you want to share about the WHO Registry Platform. |
| 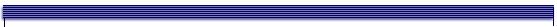 | |
| **17** | Please provide any anonymous comments you want to share about the Ottawa Statement (Parts 1 or 2): |
| 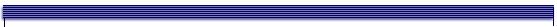 | |
|  | | First Name (Optional) |  | | --- | --- | | Last Name: (Optional) |  | | Country |  | |

Bottom of Form
